# p53 as a biomarker and potential target in gastrointestinal stromal tumors

**DOI:** 10.3389/fonc.2022.872202

**Published:** 2022-07-29

**Authors:** Chiao-En Wu, Chiao-Ping Chen, Wen-Kuan Huang, Yi-Ru Pan, Erhan Aptullahoglu, Chun-Nan Yeh, John Lunec

**Affiliations:** ^1^ Division of Hematology-Oncology, Department of Internal Medicine, Chang Gung Memorial Hospital at Linkou, Chang Gung University College of Medicine, Taoyuan, Taiwan; ^2^ Department of General Surgery and Liver Research Center, Chang Gung Memorial Hospital at Linkou, Chang Gung University, Taoyuan, Taiwan; ^3^ Department of Molecular Biology and Genetics, Bilecik Seyh Edebali University, Bilecik, Turkey; ^4^ Newcastle University Cancer Center, Bioscience Institute, Medical Faculty, Newcastle University, Newcastle upon Tyne, United Kingdom

**Keywords:** p53, MDM2, Wee1, target therapy, gastrointestinal stromal tumors (GIST)

## Abstract

KIT and PDGFRA play a major role in the oncogenic process in gastrointestinal stroma tumors (GIST) and small molecules have been employed with great success to target the KIT and PDGFRA pathways in this cancer. However, approximately 10% of patients with GIST are resistant to current targeted drug therapy. There is a need to explore other potential targets. Although p53 alterations frequently occur in most cancers, studies regarding p53 in GIST have been limited. The CDKN2A/MDM2/p53 axis regulates cell cycle progression and DNA damage responses, which in turn control tumor growth. This axis is the major event required for transformation from low- to high-risk GIST. Generally, p53 mutation is infrequent in GIST, but p53 overexpression has been reported to be associated with high-risk GIST and unfavorable prognosis, implying that p53 should play a critical role in GIST. Also, Wee1 regulates the cell cycle and the antitumor activity of Wee1 inhibition was reported to be p53 mutant dependent. In addition, Wee1 was reported to have potential activity in GIST through the regulation of KIT protein and this mechanism may be dependent on p53 status. In this article, we review previous reports regarding the role of p53 in GIST and propose targeting the p53 pathway as a novel additional treatment strategy for GIST.

## Gastrointestinal stromal tumor

GISTs are a subgroup of mesenchymal tumors arising from the gastrointestinal tract and have been considered as smooth muscle tumors (such as leiomyomas or leiomyosarcomas) based on the histologic characteristics (1). Until the discovery of KIT (CD117) expression in GIST (2), the origin of GIST was proposed to be from the interstitial cells of Cajal (ICCs) or precursors (3–5).

GISTs are relatively rare and account for 1%–2% of cases of gastrointestinal malignancy (6). They were most commonly found in the stomach (55.6%), followed by the small intestine (31.8%), colon and rectum (6%), other locations (5.5%) (7), and esophagus (<1%) in a systemic review of 9,747 GISTs. Surgical resection is the main treatment for localized GISTs. The risk of GIST recurrence was estimated using the modified National Institutes of Health consensus classification system based on the tumor size, mitotic count (8), and primary location (gastric vs. non-gastric). Most low- and intermediate-risk GISTs are cured by surgical resection. In contrast, high-risk GISTs should be treated with adjuvant therapy with 3-year imatinib, which can improve recurrence-free survival (RFS) and overall survival. Neoadjuvant therapy may be an alternative for high-risk GISTs, which can potentially increase the complete resection rate and avoid surgical rupture and resection of the involved organs (9). Palliative treatment, mainly targeted therapy, is the standard treatment for advanced/metastatic GISTs, and classical cytotoxic chemotherapy is inactive for GIST.

*c-KIT* mutations (2, 10) followed by platelet-derived growth factor receptor alpha (*PDGFRA*) mutations (11) were discovered and small molecule targeted therapies with tyrosine kinase inhibitors (12, 13) were developed in GIST. Imatinib, sunitinib, regorafenib, and ripretinib are approved in advanced GIST without selection for matched druggable targets. The *post-hoc* analysis of clinical trials found that *KIT* exon 9 mutation was associated with a lower response rate and progression-free survival (PFS) compared to exon 11 mutation under treatment with 400 mg/day of imatinib, and 800 mg/day of imatinib benefited patients with *KIT* exon 9 mutation upon progression despite 400 mg/day of imatinib (14). Although most *PDGFRA* mutations are sensitive to imatinib, D842V is resistant to imatinib and targeted agents other than avapritinib. Currently, genetic testing of *KIT* and *PDGFRA* is essential before treatment initiation (15), and comprehensive genomic profiling, such as for lung cancer, should be considered to identify driver mutations (e.g., *SDH*, *BRAF*, *NF1*, *NTRK* fusion, and *FGFR* fusion) for GISTs without *KIT* and *PDGFRA* mutations.

*KIT* and *PDGFRA* are the main driver mutations but insufficient to promote tumor progression from low- to high-risk GISTs. Additional chromosomal aberrations and subsequent dysregulation of the cell cycle are essential for tumor progression. For example, chromosome 9/9p loss, homozygous *CDKN2A* (located on chromosome 9p) deletion, p14/p16 loss, activation of MDM2/CDK4 leading to RB1/p53 inactivation, and loss of cell cycle control are considered to be the main events for progression to high-risk GISTs (16).

## Driver mutations and approved targeted therapy for GIST

The nature of GISTs became better understood with the identification of KIT (CD117) expression and *c-KIT* mutations (2, 10). Heinrich et al. investigated possible alternative receptor tyrosine kinase (RTK) oncoproteins using immunoprecipitations with polyclonal panRTK antisera and found hosphor-PDGFRA was the predominant hosphor-RTK in a *KIT* wild type GIST cell line (GIST478). Activated PDGFRA mutations were subsequently found in 35% (14 of 40) of *KIT*-WT GIST  (11). Although most GISTs have either mutation of *KIT* or *PDGFRA* kinase genes as driver mutations, approximately 10 to 15% of GISTs do not harbor a *KIT* or *PDGFRA* mutation, which are collectively grouped as *KIT*/*PDGFRA*-WT GIST.

With the understanding of the molecular biology of GIST and discovery of effective targeted therapy against KIT/PDGFRA pathways, the disease has become more manageable, but the treatments are not completely curative. Several small molecule compounds targeting KIT and other tyrosine kinases, such as imatinib (12, 13), sunitinib (17), regorafenib (18) and ripretinib (19) have shown efficacy in advanced GIST and have been approved for the treatment of advanced GIST. In the SWOG phase III S0033 trial imatinib treatment resulted in a median PFS of 18-20 months (13) and 26% of GIST patients survived 8 years or longer (20). Approximately 10% of GIST patients have primary resistance to imatinib and many of these resistant tumors lack mutations in KIT or PDGFRA, or they harbor a PDGFRA exon 18 D842V mutation. Secondary mutation on KIT (exons 13, 14, 17) developed after imatinib treatment leading to resistance to imatinib. Sunitinib and regorafenib were found to overcome such secondary mutation of KIT (21). Sunitinib treatment after failure of imatinib showed a median time to tumor progression of 27.3 weeks in GIST patients (17). Regorafenib treatment resulted in a median PFS of 4.8 months after failure of imatinib and sunitinib (18). Ripretinib produced a median PFS of 6.3 months in GIST patients after failure of imatinib, sunitinib, and regorafenib (19). All the above inhibitors are widely used in routine clinical practice for patients with advanced GIST and significantly improve the overall survival of GIST patients (22–25). In addition, for patients with symptomatic and/or rapidly progressive disease harboring a PDGFRA exon 18 D842V mutation, avapritinib has been suggested over either imatinib or observation in the setting of initial therapy. These tumors often demonstrate primary resistance to imatinib, whereas the response rate with avapritinib was nearly 90% (26).

In *KIT*/*PDGFRA*-WT GISTs, SDH deficiency and other genetic alterations, such as *BRAF*, *NF1*, and *NTRK* fusions, were the main alterations according to comprehensive genetic profiling. Therefore, the proportion of GIST without mutation is decreasing. However, quadruple WT GISTs without mutations or alterations in the *KIT/PDGFRA*, RAS pathway, or SDH complex are the current treatment challenges because of lack of active treatment for such patients and inactivity of transitional cytotoxic chemotherapy for GISTs (27).

In addition, resistance to targeted therapy is another challenge, as these tyrosine kinase inhibitors (TKIs) can cytostatically inhibitor tumor growth but not induce apoptosis leading to cell killing. Therefore, GIST may be incurable once it develops metastasis.

## Is p53 a biomarker or target in GIST?

### *TP53* in GIST

The *TP53* gene encoding the p53 tumor suppressor protein is referred to as the guardian of genome and is mutated in most human cancers, although the frequency of mutation varies according to cancer type (28). The wild-type p53 protein plays a critical role in the cellular response to DNA damage in order to induce cell cycle arrest and DNA repair, or apoptosis (28). The incidence and possible prognostic role of *TP53* mutations have been studied in most cancers (28–30). However, the role of p53 in GIST has received relatively little attention, largely because both *KIT* and *PDGFRA* deregulation play such a strong role as the major oncogenic processes in GIST (1). As CDKN2A loss, MDM2 overexpression, and p53 inactivation are the main events for tumor progression to high-risk GISTs, in this article, we review previous reports regarding p53 in GIST and propose a novel treatment strategy against GIST by non-genotoxic targeted activation of p53. In a meta-analysis including 1,163 patients from 19 studies, p53 expression was significantly higher in high-/intermediate-risk GISTs compared to low-/very-low-risk GIST and correlated with a poor prognosis (31).

Generally, *TP53* mutation has been found to be infrequent (<5%) in GIST cohorts (32–34). Henze et al, reported that *TP53* mutation was both infrequent (3%) and independent of p53 immunostaining in 62 GISTs (33). In a cohort of 83 GISTs, four patients had inactivating *TP53* or *RB1* mutations, which were associated with high-risk tumors, and three patients developed recurrent and metastatic GISTs (35).

The role of p53 alterations, other than those involving *TP53* mutation, have been evaluated in relation to GIST aggressiveness and progression. In a gene expression study of 30 localized GIST, upregulated genes were significantly enriched for those involving cell cycle regulation and the DNA damage response pathway in high risk compared to low risk GIST (36). As p53 is a key regulator responsible for cell cycle control in response to DNA damage, it may also be involved in tumor progression for high risk GIST. Other studies have confirmed that p53 expression is associated with high risk of recurrence in localized GIST (37, 38). It was reported that 40.0% of one hundred and twenty-five localized GIST had a p53 alteration, including 20.8% *TP53* mutation and 24.8% p53 overexpression, and that the GISTs with p53 alterations were more commonly found in localized high risk GISTs with shorter relapse-free survival. This suggested that p53 alteration, involving either mutation or overexpression, is a significant independent indicator of poor prognosis (36). The relatively high rate of *TP53* mutation (20.8%) in this study may be related to the high proportion of high-risk GIST (48%) (37). In another study of 96 localized GIST, p53 expression was also found to be significantly associated with increased mitotic rate and the risk of malignancy (38). Similarly, in another report of 104 KIT-positive GISTs, p53 expression significantly correlated with high-risk epithelioid GISTs regardless of the tumor site (31).

Using samples from the SSGXVIII trial (39, 40), a phase III study comparing adjuvant one-year and three-year imatinib in high-risk GIST patients, IHC was performed in 320 primary GIST and mutations analyzed using Sanger sequencing in 245 cases (41). A high expression of CDK4 (32.8%) was associated with a favorable RFS, whereas high expression of MDM2 (12.2%) or p53 (35.3%) was associated with a shorter RFS. The overall frequency of *TP53* mutations was low (3.5%) and could not be predicted by the IHC detection of p53. *TP53* mutations tend to occur more frequently in gastric GISTs associated with a worse RFS.

In a phase III study of advanced/metastatic GIST patients undergoing imatinib 400 mg vs 800 mg daily treatment, 353 GISTs were screened for p53 immunostaining, and only samples with high p53 expression were subsequently analyzed for TP53 mutations (42). Only 13 (16.4%) *TP53* mutations were found among 79 GISTs with high p53 expression, consistent with previous studies that *TP53* mutations cannot be predicted by IHC alone. In addition to previously well-known prognostic factors including performance status, *KIT* mutation, and tumor size, molecular biomarkers of low p16 and high p53 expressions were correlated with imatinib response and identified as significant and independent prognostic factors of PFS (43). Low p16 was supposed to be a result from loss of *CDKN2A* which encodes both p14^ARF^ and p16^INK4a^. p14^ARF^ is a negative regulator of MDM2 function (43).

A more comprehensive study assessed the prognostic significance of *CDKN2A*, *RB1* or *TP53* mutation/copy loss in 71 primary GISTs. The occurrence of genetic alteration was associated with high risk primary GISTs. The presence of cell cycle-related events was associated with a significantly shorter relapse-free survival (p < 0.0001) and overall survival (p = 0.042). This study provide indirect evidence that genomic alterations involving cell cycle-related genes were associated with GIST progression to malignant disease (44).

Although these results cannot be directly compared because of the different approaches, a consistent lack of close association between *TP53* mutations and IHC data was found. Alternative reasons for mutation independent activation of p53 could include cellular stress, such as hypoxia, or posttranscriptional modifications such as phosphorylation, acetylation or sumoylation.

In conclusion, *TP53* mutation is generally infrequent in GIST despite the frequency being higher in high-risk GIST. The overexpression of p53 by IHC cannot predict *TP53* mutation, but has been shown to be significantly associated with localized high-risk GIST with unfavorable relapse free survival. In addition, p53 expression was also reported to be an independent prognostic factor for advanced GIST undergoing imatinib treatment. The overall evidence suggests that p53 expression and/or *TP53* mutation play a critical role in tumor progression and may be unfavorable prognostic factors for imatinib treatment. These findings provide a rationale to explore targeting the p53 pathway as a novel therapeutic strategy in GIST.

### MDM2 alteration and as a possible target in GIST

MDM2 is transcriptionally transactivated by p53 and can negatively auto-regulate p53 by ubiquitination (28, 45), therefore MDM2 is considered an oncogene and its amplification or overexpression can increase tumor cell proliferation by suppressing p53 (46).

Regarding MDM2 amplification, only one out of 35 (3%) GISTs was found to be amplified for MDM2 in one series (47). In contrast, by IHC, 40% of GIST cases (14 out of 35 patients) were reported to be positive for MDM2 IHC staining, which was significantly associated with metastasis (48). Based on a limited study of MDM2 in GIST, MDM2 expression may be an indicator of aggressive behavior and poor prognosis, likely resulting from inhibition of p53 function. This implies that targeting MDM2 may be a potential strategy for treatment of a subset of GIST, particularly for WT-p53 GIST. Nutlin-3, an MDM2 inhibitor, has shown selectively growth inhibitory and little cytotoxic activity in WT p53 GIST cell lines (GIST430, GIST48, GIST48B) compared with p53 mutated cells (GIST882, GIST-T1) (33).

### Wee1

A study of Wee1 expression knockdown by siRNA or Wee1 kinase inhibition with MK1775 was observed to reduce KIT expression (49). This suggested that Wee1 plays a potential role in the regulation of KIT in GIST which may contribute to an anti-proliferative effect of Wee1 inhibition in this cancer. Wee1 inhibition was reported to promote the autophagic degradation of KIT and, therefore, targeting Wee1 was suggested to represent a novel strategy for GIST therapies. Another study identified a role of Wee1 in GIST using kinome profiling with loss-of-function assays, and demonstrated that in addition to *KIT* mutant GIST, Wee1 may be a promising target in PDGFRA D842V mutant GISTs (50). However, only p53 mutated cells were used in the above studies, implying this approach may be limited to p53 mutated cell lines, as indicated in previous reports of Wee1 inhibitors (28, 51, 52). It is of interest to determine whether Wee1 plays the same role in WT p53 GISTs.

## Future clinical studies targeting p53 in GIST

Targeted therapies, either alone or in combination with immune checkpoint inhibitors have been evaluated in clinical trials (53). Most studies have involved small molecule inhibitors approved in GIST, such as imatinib, sunitinib, regorafenib and/or ripretinib. However, no trials have included drugs targeting the p53 pathway, although a few preclinical studies have indicated the efficacy of such compounds.

As MDM2 expression has been associated with poor prognosis GISTSs (48) and MDM2 inhibitors (33) demonstrated to suppress growth and induced apoptosis in WT p53 GIST cells, the use of MDM2 inhibitors may be an additional strategy beyond KIT and PDGFRA in future studies. However, preclinical studies, particularly *in vivo* studies, to confirm the feasibility of using MDM2 inhibitors in GIST are lacking. As no clinical trials of p53-directed targeted therapy have been performed or are underway in GISTs, only some clinical studies on other malignancies can be summarized to highlight the applicability of such agents in GISTs in the future.

Although several preclinical studies have shown impressive results, the limited clinical trials of MDM2 inhibitors in other cancers have been disappointing. A phase III study, MIRROS (NCT02545283), failed to meet its primary goal of showing the combination therapy of idasanutlin and cytarabine was superior to placebo plus cytarabine regarding improvement of survival in acute myeloid leukaemia (54). Limited efficacy of HDM201 (siremadlin) in combination with ribociclib (a CDK4/6 inhibitor) was observed in patients with locally advanced or metastatic liposarcoma, even though this tumor highly expresses MDM2 and CDK4 (55). As no agents targeting MDM2 have so far been successful, the therapeutic strategy of targeting the p53 pathway in this way has been doubted. The next challenge is to understand why these studies have shown limited efficacy. It should be clarified whether these targeted agents insufficiently reactivate WT-p53 or if the dose limiting hematologic and gastrointestinal toxicity of p53 reactivation is not tolerated. Furthermore, the efficacy of MDM2 inhibitors may be limited by WIP1 phosphatase expression (56). WIP1 inhibition has been shown to enhance the activity of MDM2 inhibitors (28, 45, 57, 58). Unfortunately, due to the lack of suitable compounds for *in vivo* evaluation, the studies of WIP1 inhibition have been limited to the preclinical setting, however there is a strong case for the development of WIP1 inhibitors suitable for clinic evaluation.

In contrast, MK-1775 is active against p53 mutated GIST cells both *in vitro* and *in vivo*. Besides the regulation of KIT expression, synthetic lethality may be the key mechanism in p53 deficient cells (28, 51, 52). Clinical studies of MK-1775 are ongoing and early phase studies in various tumors have reported encouraging results (59–62).

RITA, a p53 activator, induced p53 in only one p53-WT GIST48B (but not GIST430 or GIST48), followed by growth inhibition and apoptosis. GIST-T1, a p53-mutated cell line, was also highly sensitive to RITA without p53 activation. The aforementioned findings suggested the feasibility of a p53-independent mechanism of action for RITA treatment (24). As no biomarkers can predict the response or resistance to RITA, RITA may not be a good targeted agent in GIST.

## Proposed incorporation of targeting based on p53 status into current GIST clinical practice

**Figure 1:**
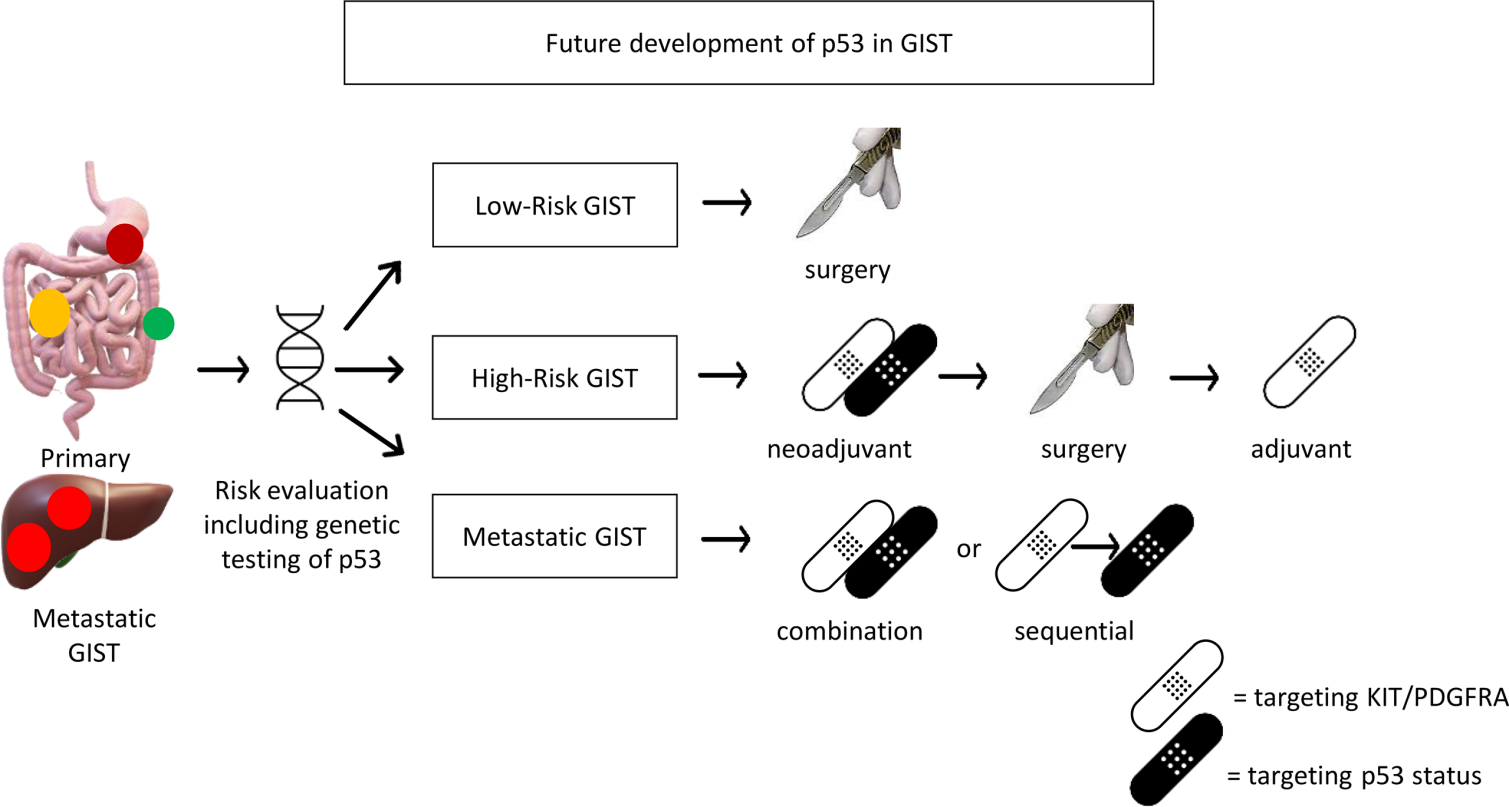
Future development of p53 for potential clinical application in GIST. Genetic testing including p53 should be included for risk evaluation. p53 targeted therapy (MDM2 inhibitor for wild-type p53 or Wee1 inhibitor for mutant p53) may be incorporated in palliative treatment for metastatic GIST or neoadjuvant treatment for high-risk GIST (if it works in metastatic GIST).

Based on the above evidence, CDKN2A/MDM2/p53 pathway alterations frequently occur in high-risk GISTs. The clinical application of this knowledge may be a prognostic factor, or a predictive factor to identify patients who would benefit from adjuvant imatinib treatment (63). Therefore, the genetic testing of these p53 pathway components is required and p53 status would be a critical biomarker for risk stratification beyond the current model based on clinical features (location, tumor size, mitosis). In addition, targeted treatment based on p53 status, either as monotherapy or in combination with KIT/PDGFRA targeted therapy, is proposed for metastatic GISTs (**Figure 1**). Furthermore, if p53-directed targeted therapy shows promising results in metastatic GISTs, the incorporation of p53-directed targeted therapy may be considered for development in the neoadjuvant setting for high-risk GIST, in combination with KIT/PDGFRA targeted therapy to increase response rate and maximize tumor shrinkage (**Figure 1**). However, due to currently limited studies as described above, preclinical research is warranted to build a case for the evaluation of such a treatment strategy in clinical trials.

## Conclusions

**Figure 2:**
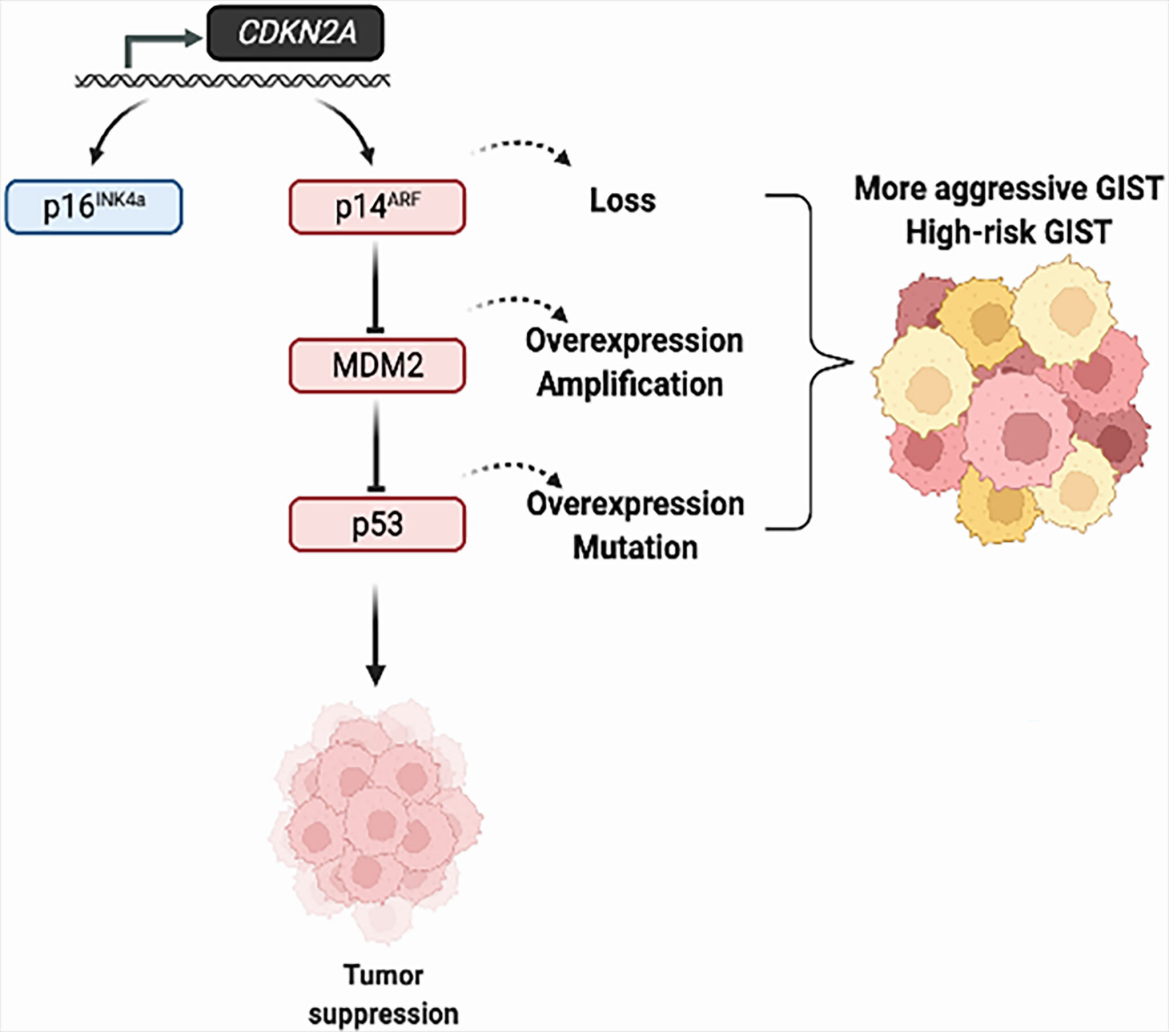
Alterations of the CDKN2A/MDM2/p53 pathway in GIST tumors. The tumor suppression function of p53 is regulated by MDM2 and p14ARF. The overexpression of MDM2 and loss of p16^INK4a^/p14^ARF^, which in turn influence p53 function, lead to tumor progression in GIST.

CDKN2A/MDM2/p53 pathway alterations in GIST have been associated with high-risk GIST, adverse clinicopathological characteristics and poor patient survival (**Figure 2**). Preclinical studies have demonstrated the potential activity of MDM2 inhibitors in p53-WT GIST cells and Wee1 inhibitors in p53-mutated GIST cells. Clinical trials are warranted to determine the role of these novel targeted treatments in GIST.

## Author contributions

Conceptualization, C-EW, W-KH, C-NY, and JL. Data curation, C-EW and C-PC. Formal analysis, C-PC and Y-RP. Funding acquisition, C-EW and C-NY. Investigation, EA. Methodology, C-EW, and Y-RP. Project administration, C-EW. Supervision, JL. Validation, C-EW, W-KH, C-NY, and JL. Visualization, C-PC and EA. Writing—Original draft, C-EW. Writing—Review and editing, C-NY and JL. All authors contributed to the article and approved the submitted version.

## Funding

This research was funded by Linkou Chang Gung Memorial Hospital, grant numbers CMRPG3J0971~3, NMRPD1K1332~3, CMRPG3L0911 to C-EW.

## Acknowledgments

This work was supported by grants from Linkou Chang-Gung Memorial Hospital and the Ministry of Science and Technology in Taiwan. Please refer to funding section.

## Conflict of interest

The authors declare that the research was conducted in the absence of any commercial or financial relationships that could be construed as a potential conflict of interest.

## Publisher’s note

All claims expressed in this article are solely those of the authors and do not necessarily represent those of their affiliated organizations, or those of the publisher, the editors and the reviewers. Any product that may be evaluated in this article, or claim that may be made by its manufacturer, is not guaranteed or endorsed by the publisher.
